# Local environmental attributes and type 2 diabetes: Green amenities, walkability indicators, and air pollution are associated with incidence

**DOI:** 10.1016/j.envint.2025.109870

**Published:** 2025-10-17

**Authors:** David S. Curtis, Huong D. Meeks, Ken R. Smith, Kyle Kole, Barbara B. Brown, Sara Grineski, Austin S. Clark, Lori Kowaleski-Jones

**Affiliations:** a Department of Family & Consumer Studies, University of Utah, Salt Lake City, UT 84112, USA; b Department of Pediatrics, University of Utah, Salt Lake City, UT 84112, USA; c Department of Sociology, University of Utah, Salt Lake City, UT 84112, USA; d School of Environment, Society and Sustainability, University of Utah, Salt Lake City, UT 84112, USA

**Keywords:** Type 2 diabetes, Greenness, Pollution, Walkability, Fast food, Environment, Neighborhood effects

## Abstract

Although multiple environmental attributes have been associated with type 2 diabetes (T2D) incidence, longitudinal evidence is needed that accounts for co-exposures and key confounding variables. This study estimates associations between environmental attributes (i.e., green amenities, air pollution, neighborhood walkability indicators, and availability of food retailers) and T2D incidence. Records came from the Utah Population Database and included individuals born from 1970 to 1990 and their parents residing in the four-county urban core of Utah (*N* = 909,729). T2D diagnoses came from multiple health data sources, spanning from 1996 to 2019. Time-varying environmental attributes were objectively measured, and covariate adjustment included area- and individual-level socioeconomic characteristics, geographic correlates of environmental attributes, and family history of T2D. Results from Cox regression models indicated that higher block group-level residential greenness (HR = 0.945, 95% CI: 0.92, 0.97), green land cover (HR = 0.984, 95% CI: 0.97, 1.00), and active commuting rate (HR = 0.964, 95% CI: 0.95, 0.98) were independently associated with reduced T2D hazard. In contrast, higher concentration of ambient particulate matter (HR = 1.079, 95% CI: 1.04, 1.12) and intersection density (HR = 1.026, 95% CI: 1.01, 1.04) were associated with increased T2D hazard. Population density and food retailer availability were not associated with T2D hazard. Inferences were similar in sensitivity tests that aimed to remove prevalent cases, accounted for period effects, and modeled attributes at the scale of census tracts. Our findings suggest that green amenities, air pollution, and land use should not be overlooked in societal efforts to improve population-level metabolic health.

## 1. Introduction

From 1990 to 2010, type 2 diabetes (T2D) incidence in the United States increased by nearly 50 percent (Selvin et al., 2014). This rise is commonly attributed to shifts in dietary patterns and more sedentary lifestyles—behaviors that are shaped by the broader environment (French et al., 2001; Howell and Booth, 2022). Changes in residential environments therefore are potential contributors to rising T2D, and prior research has reported associations between local environmental attributes (e.g., residential greenness, neighborhood walkability) and T2D risk (den Braver et al., 2018; Dendup et al., 2018). Much of this research, however, has relied on cross-sectional designs or a single measurement of exposures (Chandrabose et al., 2019; den Braver et al., 2018), and has typically examined only a narrow set of environmental attributes (Dendup et al., 2018; Yang et al., 2020). Therefore, the extent to which environmental attributes influence T2D remains uncertain.

### 1.1. Environmental attributes as predictors of type 2 diabetes

The local residential environment may influence T2D risk through multiple pathways, such as supporting physical activity, facilitating social interactions and stress coping, and being the source of harmful physical exposures (Bird et al., 2018; Markevych et al., 2017). Environmental attributes with potential to influence health behaviors include greenspaces, walkable streets, and availability of grocery stores and fast food retailers (Frank et al., 2019; Klitzman et al., 2006; Markevych et al., 2017). Such spaces also provide physical locations for social encounters and gatherings that can foster social cohesion (de Vries et al., 2013). In contrast, local environmental harms include the concentration of air pollutants and traffic-related noise (Sørensen et al., 2022). Different types of environmental attributes are interdependent and tend to be correlated with resident characteristics (e.g., household and area socioeconomic conditions) (Althor and Witt, 2020; Curtis et al., 2024), such that relationships with health may be complex. For instance, despite higher population density generally indicating increased walkability, dense urban development may bring disamenities and environmental harms (e.g., air pollution, noise, crowding), particularly when not paired with amenities such as greenspace (McDonald et al., 2023; Son et al., 2021). Thus, density-related urban amenities (e.g., walkability) can come at the cost of declining nature contact and rising air pollution if greenspace is replaced with physical infrastructure and automobile-oriented street design (Clark et al., 2017; Park and Guldmann, 2020). Given varied processes linking place to health and interdependencies between environmental attributes and area features, research needs to thoroughly account for co-exposures to reduce bias in estimated associations between environmental attributes and health (Frank et al., 2019; Howell and Booth, 2022; Sørensen et al., 2022).

Mixed but generally supportive findings exist for higher residential greenness predicting reduced T2D risk (den Braver et al., 2018). Evidence is of similar quality for greenness predicting potential intermediary outcomes (i.e., physical activity, lower obesity, reduced low-grade systemic inflammation) (Browning and Lee, 2017; Fong et al., 2018). Although the majority of evidence is cross-sectional (Dendup et al., 2018), recent longitudinal studies support residential greenness as a protective factor for T2D incidence (Doubleday et al., 2022; Tsai et al., 2021).

Meta-analyses find that greater long-term exposure to ambient air pollution (e.g., particulate matter of ≤ 10 μm or ≤ 2.5 μm) is associated with increased hazard of T2D (Liu et al., 2019; Yang et al., 2020). However, studies on air pollutants and T2D typically do not account for built environment characteristics (e.g., walkability) that correlate with air pollution—although adjustment is commonly made for greenness, noise, and physical toxins (Son et al., 2021; Yang et al., 2020). For example, one study assessed co-exposure to four air pollutants, finding all predicted T2D incidence but that associations were attenuated when adjusting for greenspace and traffic noise (Sørensen et al., 2022).

Greater neighborhood walkability has been associated with reduced T2D risk (den Braver et al., 2018), possibly by influencing physical activity and social cohesion (de Vries et al., 2013; Frank et al., 2019). Although longitudinal evidence exists for walkability as a protective factor for T2D (Chandrabose et al., 2019), most studies included a single temporal measurement of walkability and few directly adjusted for self-selection of more active participants into walkable neighborhoods. Moreover, research on walkability and T2D frequently does not account for key area-level confounds, such as greenspace, pollution, and socioeconomic circumstances (Dendup et al., 2018).

Whether the local food environment influences T2D risk, likely through dietary quality, is uncertain (Allcott et al., 2019; den Braver et al., 2018). In particular, new supermarkets in a community do not appear to alter dietary habits (Allcott et al., 2019), yet some observational studies have reported improved health in areas with more grocery stores and fewer fast food outlets (Black et al., 2014; Fan et al., 2014; Zick et al., 2023).

### 1.2. Current study

The aim of this study is to estimate the association between multiple environmental attributes and T2D incidence. We focus on green amenities, ambient air pollution, neighborhood walkability indicators, and two types of food retailers, modeling their independent associations with T2D. These attributes have been linked to T2D in prior research (Frank et al., 2019; Sørensen et al., 2022), yet much of the literature uses cross-sectional data or a single measurement of area attributes and lacks sufficient adjustment for individual- and area-level covariates. To address such limitations, we include time-varying measures spanning from 1995 to 2015 that allow for residential mobility and changing area conditions. Additionally, we account for potential confounding variables: area- and individual-level socioeconomic and demographic characteristics that relate to residential patterns and health; geographic variables that relate to the patterning of amenities; and familial history of T2D. This study uses a unique database that includes state administrative and health records for youth and adults in the four-county urban core of Utah and information on their residential histories and family history of T2D.

## 2. Methods

### 2.1. Study population

**Fig. 1.**
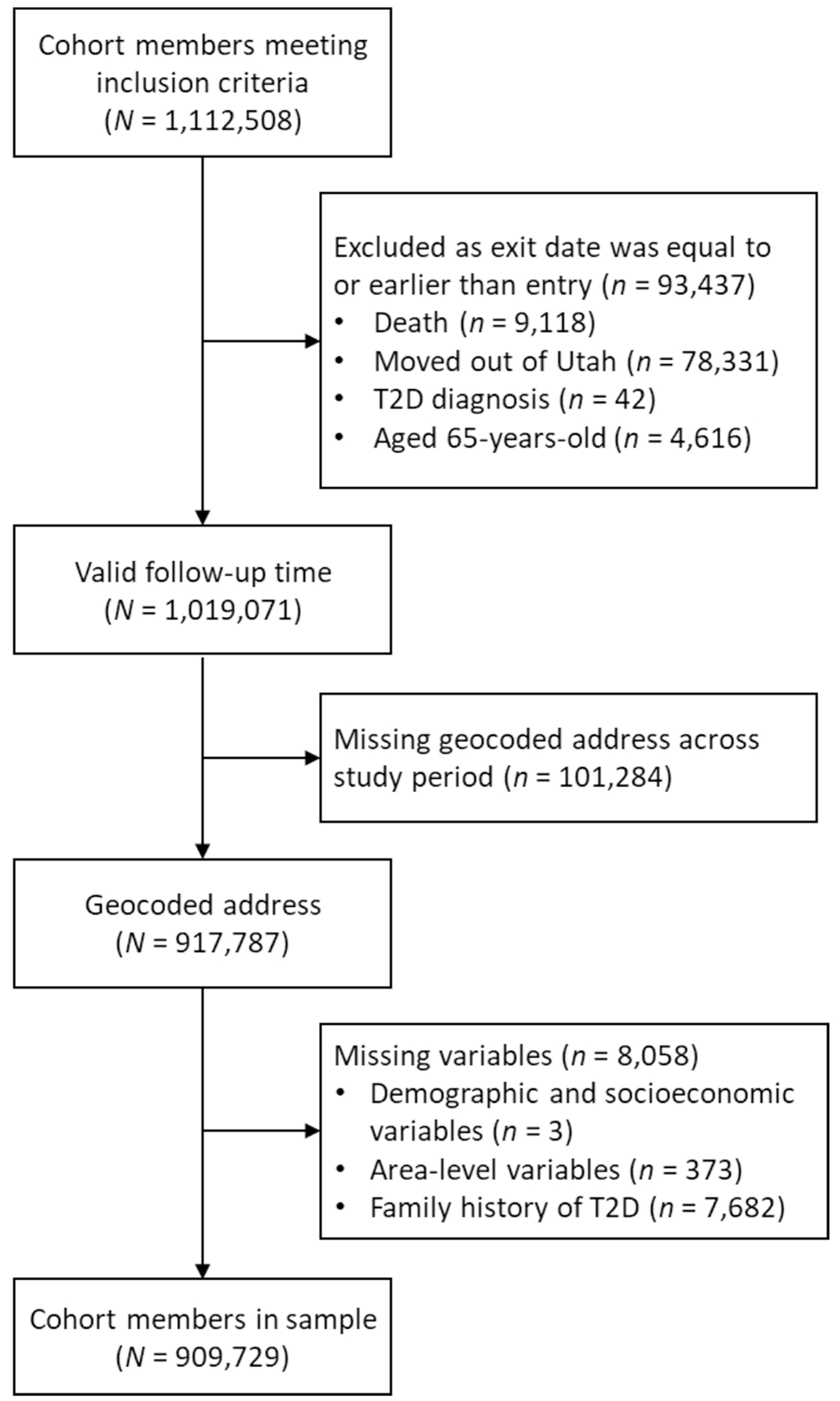
Flow diagram for offspring and parent cohorts in four-county urban core in Utah, derived using records from the Utah Population Database for 1996 through 2019.

Data came from the Utah Population Database (UPDB), a rich source of health and demographic records covering the Utah population appearing in statewide administrative records (Smith and Mineau, 2021). The UPDB is derived from Utah state records, such as vital statistics, marriage and divorce certificates, and driver licenses, and is linked to medical information from numerous sources (e.g., state-licensed hospitals). We included two related cohorts: 1) individuals born between 1970 and 1990 who resided in a four-county urban area in Northern Utah at any point between 1990 and 2015, and 2) the parents of these individuals who appeared in the UPDB. The inclusion criteria for this study stem from a project on the intergenerational transmission of T2D among young adults which included an offspring cohort and their parents (Smith et al., 2024). The requirement to reside in the study area was to ensure availability and comparability of environmental attributes. Of the cohort members meeting the inclusion criteria, individuals were excluded when they exited the risk pool before being eligible for observation (e.g., due to moving outside of Utah prior to the availability of health records), if they did not have a geocoded address across the study period, or if missing data on covariates. We show the steps of selecting cohort members in Fig. 1. The analysis included 909,729 individuals with 15,240,973 person-years at risk. This study was approved by the University of Utah Institutional Review Board (IRB #00108522) and the Resource for Genetic and Epidemiologic Research, a regulatory body overseeing access to UPDB.

### 2.2. Study area and area definitions to identify exposure

The four-county study area (i.e., Weber, Davis, Salt Lake, Utah counties) comprises the urbanized portion of Ogden-Clearfield, Salt Lake City, and Provo-Orem metropolitan statistical areas. Residential census block groups and tracts denote geographic areas for the linkage of environmental attributes. Census block groups, in urban areas, can approximate the walkable neighborhood and residential environmental exposure (e.g., air pollutants). Block groups in the current study had a median land area of 0.84 square km and an interquartile range from 0.55 to 1.61 square km (*n* = 1249). However, tracts may be the preferred geography for the food environment given the likelihood of driving to grocery stores and restaurants (Fan et al., 2014). The median land area of tracts was 2.89 square km (*n* = 439). We use 2010 census geographic boundaries for all measurement periods (Logan et al., 2016). For variables derived from population estimates (e.g., 2000 decennial census estimates), we applied interpolation weights from the National Historical Geographic Information System (NHGIS) to harmonize estimates using 2010 boundaries.

The residential history of study subjects spanned from 1993 through 2017 and included residential address information as collected through voter registrations, driver license applications, and vital statistics. Thus, data completeness and frequency of updates depends on interactions with sources. Addresses were geocoded using 2010 census boundaries by UPDB staff, with block group and tract geocodes for all known addresses within the four-county area available for this study. Of all available geocodes, 88% were classified as high confidence geocodes as all necessary address elements were present while another 7% were classified as having only minor discrepancies. We determined residential geocodes corresponding to five approximate time points: 1995, 2000, 2005, 2010, and 2015. First, we selected the geocodes corresponding to the most recent addresses prior to and on/after the time point of interest. When identical, we used this geocode for the period. When different, we used the geocode corresponding to the most proximate time point within two years. In the case of a tie, the geocode for the address prior to the time point was used. Missing geocodes were imputed for the offspring cohort when individuals were younger than 19-years-old by using the maternal and then paternal residential geocode for each period. Individuals were excluded when missing geocodes across the entire period, although we note this may have resulted from residence outside the four-county area (rather than the absence of address information in UPDB).

These five time points were used to define environmental exposures and model T2D diagnoses for the following five-year period (e.g., environmental attributes for the 2000 geocode were linked to T2D incidence from 2000 through 2004). For environmental attributes exhibiting relatively higher year-to-year variation (e.g., air pollution), we opted for two-year averages to approximate more stable exposures. For variables available at only ten-year intervals, we interpolated the intermediate time points by averaging surrounding measurements. Thus, exposures were assigned at the start of the follow-up period, ensuring that exposure preceded T2D diagnosis.

### 2.3. Measures

Study variables, data sources, and the timing of measurement are summarized in Table 1.

#### 2.3.1. T2D incidence

T2D diagnoses were observed from November 1995 through December 2019 using diagnostic codes from three data sources linked to the UPDB, primarily from encounters at health facilities as well as insurance claims and secondarily from multiple cause-of-death mortality records. We identified T2D diagnoses using ICD-9 and ICD-10 codes from these sources (i.e., 250.X0 and 250.X2 for ICD-9 and E11.XXX for ICD-10; Dugan and Shubrook, 2017; Weng et al., 2016), with the timing of incident cases identified using month and year of first recorded diagnosis. This approach only captures the first T2D diagnosis in the included data sources, which is not necessarily an individual’s initial diagnosis. However, given the relatively young age of the sample in 1995, we expected the first recorded diagnosis would be a good approximation of age at diagnosis. Additionally, in sensitivity tests, to increase the likelihood of observing incident cases, we used the first three years of observation for individuals to identify prevalent T2D cases and excluded any individuals who received a T2D diagnosis during this period.

As the first data source, the Healthcare Facility Database includes encounters for all licensed hospitals, emergency rooms, and ambulatory surgery centers in Utah (Utah Department of Health and Human Services, 2017). Reporting requirements for these facility types were in effect for the study period from the end of 1995 through 2019. Although the types of observed encounters are limited to these facilities, such that the diagnosis may come later in the course of T2D as compared to encounters in primary care, this database includes encounters regardless of insurance coverage. Second, the Utah All Payer Claims Database (APCD) contains insurance claims from commercial health insurance carriers enrolling at least 2,500 individuals and from Medicaid. Although Medicare-covered claims are generally not included in the APCD, Medicare Supplement and Medicare Advantage claims may be reported when provided by commercial insurers. The share of Utah residents in the APCD varies over the study period, with insurance claims first reported in 2009 and substantially increasing by 2013. In our cohorts, among those with a recorded address circa 2015, 75–80% of individuals had insurance plans in the APCD for individual years from 2013 through 2016, decreasing to ~ 64% in 2017 through 2019, with over 90% of individuals in the APCD for at least one year from 2015 to 2019. The decrease in coverage of insured individuals in the Utah APCD after 2016 resulted from a Supreme Court ruling exempting self-funded employee health plans from state APCD reporting requirements (Brown and King, 2016). Third, multiple cause of death data were available from death certificates for Utah decedents, and T2D diagnosis was coded if listed as primary or secondary cause of death. Altogether, with the inclusion of the Healthcare Facility Database, APCD, and mortality records, we expect to capture the vast majority of diagnosed T2D cases among the eligible cohorts. Even so, ascertainment of T2D depends on participant use of health care, such that differences in access to health care may bias estimates of time to T2D. Additionally, for the period before the inclusion of insurance claims from the APCD, ascertainment of T2D may come later in the course of T2D, such that we adjust all models for a binary variable representing the period when APCD records were relatively complete (i.e., approximated as after August 2012).

#### 2.3.2. Green amenities

We included residential greenness and green/natural land cover as green amenities. Residential greenness is assessed using the summertime normalized difference vegetation index (NDVI), a satellite-derived measure of vegetation levels estimated using the red and near-infrared bands of the electromagnetic spectrum. Landsat 5 and 8 images (Collection 2, Tier 1, Level 2) with a pixel resolution of 30 m were obtained through Google Earth Engine for June through August in two-year periods corresponding to quinquennial assessments (Gorelick et al., 2017). Only pixels with cloud cover of less than 10% and positive NDVI values (negative values indicate water) were used when calculating average NDVI in each block group. To assess the share of green or natural land cover, we used primary land cover classifications from Land Change Monitoring, Assessment, and Projection (LCMAP) Analysis Ready Data for 1992, 2001, and 2011 (Brown et al., 2020). For periods corresponding to the midpoint between assessment (e.g., 2005), we took the average of the surrounding assessments. Given the semi-arid climate of the region and potential for different types of nature exposure to influence health (Li et al., 2023; Nazif-Munoz et al., 2020), we included grass/shrub, tree cover, wetland, water, and barren land classifications when determining the share of green or natural land cover. Hereafter we refer to this measure as green land cover because grass/shrub and tree cover are the predominant types, accounting for more than 75% of the total land cover captured in our category of green/natural.

#### 2.3.3. Air pollution

We included two-year averages of particulate matter with an aerodynamic diameter of 10 μm or less (PM_10_) to measure ambient air pollution in each period. Block group estimates were obtained from the Center for Air, Climate and Energy Solutions (CACES) v1 empirical models, which integrate data from air quality monitoring networks, land use regression, and satellite-derived air pollution measurements, with block group values population-weighted (Kim et al., 2020). Although other air pollutants also are risk factors for T2D, we used PM_10_ for several reasons: (1) CACES estimates for PM_10_ were available across the study period whereas PM_2.5_ estimates began only in 1999; (2) PM_10_ has a similar association with T2D as PM_2.5_ and NO_2_ (Yang et al., 2020); (3) PM_10_ was moderately to strongly correlated with other pollutants, such that inclusion of multiple pollutants could introduce multicollinearity problems and complicate interpretation (e.g., PM_10_ and NO_2_ exposure was correlated at 0.77); and (4) PM_10_ captures a broad range of PM, from ultrafine to coarse. Our aim is therefore to capture overall PM exposure rather than pollutant-specific associations, and results should be interpreted accordingly.

#### 2.3.4. Neighborhood walkability

As indicators of walkable neighborhoods, we included population density, intersection density, and the active commuting rate within block groups (Curtis et al., 2024; Smith et al., 2008). These measures are informed by the three Ds of walkability: density, pedestrian-friendly design, and diversity of land uses, respectively (Cervero and Kockelman, 1997). Although prior research typically has used a composite measure to represent walkability (Watson et al., 2020), we modeled indicators separately to identify how specific walkability dimensions were associated with T2D. Identifying the most health-relevant walkability dimensions may better inform urban design guidelines as compared to research using multi-dimensional composite measures (Chandrabose et al., 2019).

Population density proxies for walkability as a higher density of individuals attracts amenities and walkable destinations, such as public transportation, stores, and jobs. Population density is calculated as the total population in 1,000s per square kilometer of land area using population counts from 1990, 2000, 2010, and 2020 decennial censuses (Manson et al., 2022). Intersection density is typically used to assess the walkability of a street network, as interconnected streets can improve the efficiency of walking routes (Fonseca et al., 2022). Intersection density was derived using Topologically Integrated Geographic Encoding and Referencing (TIGER) Census data for 1992, 2000, and 2010 (US Census Bureau, 2021). After excluding interstate highways and dirt roads, the number of street intersections with three or more links was calculated for each block group and divided by land area in square kilometers. The active commuting rate is a population-level behavioral measure of walkability, as active commuting is likely higher in areas where homes are near places of work (Quinn et al., 2017). This was assessed as the percentage of workers who walk, bike, or use public transit to commute to work, using data from the 1990 and 2000 decennial censuses and the American Community Survey 2006–2010 and 2013–2017 five-year estimates (Manson et al., 2022).

#### 2.3.5. Food environment

We included tract-level availability of two broad types of food retailers: 1) fast food and convenience stores, which predominantly offer highly processed energy-dense food options, and 2) supermarkets, grocery stores and full-service restaurants, which typically offer more varied food options that include unprocessed and fresh foods (Farley et al., 2009). We note that the classification of full-service restaurants within this typology is not straightforward. Prior research shows consumption at both fast food and full-service restaurants is associated with higher daily intake of total energy, saturated fat, and sodium; however, fast food consumption was otherwise uniquely linked with a nutrient profile that may increase T2D risk, including higher sugar and reduced fiber, magnesium, and vitamin D intake, while full-service restaurant consumption was associated with greater intake of omega-3 fatty acids and potassium (An, 2016). Our categorization of full-service restaurants also was influenced by findings showing their greater likelihood to offer healthy food options and that access has been linked with produce consumption (Black et al., 2014; Larson et al., 2009). Counting food retailers is a common approach when characterizing the food environment (Caspi et al., 2012). Data on store locations for 1990, 2000, and 2010 came from Dun & Bradstreet (Dun and Bradstreet, 2010). We followed recommendations for data cleaning that involved correcting inconsistent classification of SIC codes (e.g., for national chains), removing addresses where locations were approximated using ZIP codes, and eliminating duplicates (Jones et al., 2017).

#### 2.3.6. Standard covariates

Participant demographic and socioeconomic characteristics were included as covariates due to residential patterns and their association with T2D prevalence. As demographic variables, we included time-varying age (in years), biological sex, race and ethnicity (non-Hispanic [NH] White; Hispanic, any race; NH American Indian/Alaska Native; NH Black; NH Asian; NH Native Hawaiian and Other Pacific Islander; NH multiracial; and unknown), and time-varying marital status (single or unknown, married, separated/divorced, widowed). For socioeconomic characteristics, we included most recent educational attainment through 2015 (less than high school, high school diploma, some college, four-year college degree, graduate or professional degree, unknown). This timing was selected to account for adulthood educational attainment, and assessments early in life would be age-biased as attainment is ongoing. Although the measurement timing introduces potential for reverse causality (T2D influencing attainment), we expected the relationship to primarily flow from education to T2D prevention.

We included number of comorbidities as a time-varying covariate, assessed at the beginning of each five-year period, to identify associations between environmental attributes and T2D independent of other health conditions. In particular, number of comorbidities may indicate greater health care use, thus reducing bias due to differential likelihood of T2D ascertainment, and proxy for generalized physiological dysregulation or disease risk. We used a modified 15-item version of the Charlson Comorbidity Index that weights specific diseases based on mortality risk and sums these weights after removing diabetes from the index (Quan et al., 2011). Disease diagnoses came from the same health data sources as T2D.

Geographic differences in diabetes rates partially arise from the socioeconomic composition of residents, as households are segregated by socioeconomic status and socioeconomic status disparities in diabetes exist (Cheng et al., 2019; Reardon et al., 2015). Adjustment for individual- and area-level socioeconomic covariates also may reduce bias from residential self-selection, as socioeconomic characteristics influence residential patterns (e.g., due to economic constraints and preferences) (Curtis et al., 2024; McCormack and Shiell, 2011). To measure neighborhood socioeconomic status, we averaged five standardized block group indicators: inflation-adjusted per capita income for persons at least 15 years old; proportion of households living at or below 150% of the federal poverty level (reverse-coded); homeownership rate; and educational attainment for adults aged at least 25 years (percentage attaining less than high school diploma [reverse-coded], and percentage with 4-year college degree) (Curtis et al., 2024). Data came from the 1990 and 2000 decennial censuses and American Community Survey 2006–2010 and 2013–2017 five-year estimates obtained from NHGIS (Manson et al., 2022). In addition, block group racial and ethnic composition was proxied using the percentage of residents identifying as non-Hispanic White (NHW). Race and ethnicity estimates came from the 1990, 2000, 2010, and 2020 decennial censuses, with the decennial census selected for all years to avoid sampling bias (Manson et al., 2022).

#### 2.3.7. Additional covariates to account for residential selection and familial risk

The patterning of area (dis)amenities may be place-specific due to topographic features, historical urban development, proximity to the city center, and local government policy—with such factors also influencing residential sorting processes (Brueckner et al., 1999). For example, the eastern side of the study region contains mountain foothills and a disproportionately affluent and White population, while the west side experiences relative social, economic, and environmental disadvantage (Curtis et al., 2024). Moreover, an interstate freeway runs north–south through the four counties, while the Salt Lake and Ogden metropolitan areas have additional freeway systems, with much of the industrial development, shopping centers, and automobile traffic being concentrated nearby. The interstate freeway also is a key source of vehicle noise pollution. Thus, as potential geographic confounders, we included longitude coordinates (to account for the east–west health gradient), distance to interstate freeway, distance to city hall in the nearest metropolitan area primary city (proxying for distance to city center; Holian, 2019), and county fixed effects. These variables were included as time-invariant measures but linked using residential geocodes at each time point to account for moves.

Familial history of T2D was used as a covariate to account for the strong heritability of T2D (Willemsen et al., 2015), and because genetic and family environment influences have been found for propensity to reside in urban settings (Duncan et al., 2012; Maxwell et al., 2021). Thus, if familial health history correlates with factors influencing residential selection (e.g., propensity for urban residence, personality and cognitive factors), adjustment for familial history of T2D also may partially reduce bias from residential selection. To assess familial T2D history, we calculated the number of T2D cases among eligible relatives as a function of total years at risk of observing T2D, weighted using the kinship coefficient for eligible relatives (Smith et al., 2024). Relatives up to three degrees of relatedness were considered eligible if residing in Utah as an adult between 1996–2019. T2D cases were observed for relatives from 1996 through 2019 using the same health data sources and procedures as the study cohorts. Years at risk for eligible relatives was calculated based on number of years alive, aged at least 18, and residing in Utah from 1996 to 2019 while not diagnosed with T2D. The kinship coefficient approximates genetic relatedness, with first-degree relatives having a coefficient of 0.5, second-degree relatives of 0.25, and third-degree relatives of 0.125. The variable was scaled so a 1-unit change equaled 1 diabetes case per 100 years at risk among kinship-weighted relatives and censored at 10 to reduce the influence of extreme outliers. The quantity of familial information varied across records, such that we included an indicator representing whether an individual had family members at risk for at least 100 cumulative years (79% of study subjects met this criterion). This indicator was interacted with familial T2D history in models as we assumed that the familial T2D history measure would have greater validity for individuals with more familial data.

### 2.4. Analysis

We used Cox regression to model associations between environmental attributes and time to T2D diagnosis. In particular, we fit the following series of models: first, separately modeling the association between each environmental attribute and T2D incidence, without adjustment for other attributes (Model 1); second, jointly modeling all attributes to estimate their independent associations (Model 2); third, adding geographic variables that relate to the patterning of environmental attributes (Model 3); and finally, adding familial history of T2D (Model 4). All models included individual- and area-level socioeconomic and demographic covariates, listed in subsection 2.3.6, as these are commonly included covariates in this literature. Geographic and family history covariates were added in subsequent models to examine how coefficients changed in response. We examined Variance Inflation Factors (VIF) to quantify multicollinearity for the environmental attributes. Although no absolute VIF threshold exists to indicate multicollinearity problems, 5 and 10 are commonly cited (O’Brien, 2007). In Model 2, the only environmental attribute above 3 was population density (VIF = 3.13). In Model 3, population density (3.19) and PM_10_ (3.20) had the highest VIF coefficients whereas other environmental attributes were below 3. Thus, the results did not indicate likely multicollinearity problems. All analyses pooled the offspring and parent cohorts and adjusted for differences between cohorts.

The origin time when participants became at risk was defined as the latter of November 1995 (i.e., beginning of the Healthcare Facility Database) or first residence in Utah (to account for late entry into the risk pool). For participants not receiving a T2D diagnosis during the observation period, exiting the risk pool (i.e., right censoring) was determined based on the earliest of: death, moving outside of Utah, turning 65 years old, or December 2019. These events were chosen because death prevents a future T2D diagnosis, most UPDB data sources include only Utah residents, many Medicare plans were not reported to APCD, and to maintain a similar follow-up period relative to the quinquennial area measurements (while avoiding the COVID-19 pandemic). We modeled time-varying covariates using an episode-splitting approach where variables, including environmental attributes, were defined at the beginning of five-year periods while time to T2D diagnosis was still observed on a monthly scale. Five-year periods were used for environmental exposures as a higher temporal resolution was not available for most measures. We also expected that residential moves would be limited and that a few follow-up years may be necessary to observe associations between exposures and T2D hazard. Individuals were excluded from the risk pool for the periods with missing data for time-varying variables but included for periods with available data. Among the analytic sample eligible to be included at each period (i.e., met study inclusion criteria, resided in Utah, not yet exited the risk pool), 78% had data in 1995 and 2000, 84% in 2005, 88% in 2010%, and 90% in 2015—and thus these individuals contributed to the estimation of hazard ratios for those periods. However, the vast majority of records excluded by period (approximately 97%) were because of a missing geocode; many of these were likely because the individual lived outside the four-county area for which we received addresses rather than missing an address in the study area.

Cox regression assumes proportional hazards for modeled covariates and employs a partial likelihood function that does not make parametric assumptions of the hazard function (Cox, 1972). We assessed the proportional hazards assumption using Schoenfeld residuals. To fit Cox models, we used the stcox command in Stata/BE 18.0 with the breslow and vce(cluster) options. The Breslow method addresses ties in the data, approximates the exact marginal likelihood, and treats time as continuous. Although we observed events at a monthly interval, we assumed time was continuous because the long observation period (i.e., up to 24.1 years or 290 months) allowed for a reasonable approximation. Robust standard errors were estimated to account for the clustering of observations within census tracts. The clustering of individuals within families was not considered due to the complex cross-classified structure where both sibling relationships and parent–child triads exist simultaneously while siblings do not nest under the same parents. However, adjustment for familial history of T2D captures a key source of statistical dependence between families.

We conducted sensitivity tests to examine the robustness of model results. First, to increase the likelihood of observing incident cases, we specified the origin time as three years after participants became at risk and excluded those who received a T2D diagnosis during their first three years of being observed. Second, as temporal trends in both environmental exposures and the risk of T2D diagnosis may bias estimates, we added period fixed effects that corresponded to the time-varying environmental exposure measurements. Third, to examine the scale effect of the modifiable areal unit problem, we modeled tract-level instead of block group-level environmental attributes. Finally, we modeled time to T2D diagnosis separately for the offspring and parent cohort.

## 3. Results

Sample descriptive statistics are presented in Table 2. These statistics are shown separately for the offspring and parent cohort, but subsequent analyses are pooled across cohorts. For the offspring cohort, the mean age when first being observed (entering the risk pool) was 15.0 years, and the average follow-up period was 20.4 years, whereas the parent cohort was first observed at an average of 41.2 years with 17.6 years of follow-up. The rate of T2D diagnosis during the study is 3.8% for the younger offspring cohort relative to 16.6% for the parent cohort. Descriptive statistics summarizing time-varying exposure to environmental attributes by five-year periods are presented in Table 3 and correlations between time-varying environmental attributes are shown in Table S1. The maximum standardized mean difference (SMD) between any two periods is reported to document temporal changes in environmental attributes. These results refer to mean exposure levels among the sample included at any point in the five-year interval, and indicate very large differences in PM_10_, small differences in green amenities and food retailer density indicators, and very small differences in walkability indicators.

Cox regression results for associations between environmental attributes and T2D hazard are shown in Table 4. Estimates in Model 1 came from separate models for each environmental attribute after adjusting for individual and block group covariates. Both block group-level green amenities are significant protective factors, with higher residential greenness and more green land cover associated with lower hazard of T2D. In contrast, higher ambient concentration of PM_10_ in block groups is associated with increased hazard of T2D. For the block group walkability indicators, both population density and intersection density are associated with higher T2D hazard, whereas the active commuting rate was associated with reduced T2D hazard. Finally, neither fast food/convenience stores nor grocery/full-service stores in tracts was associated with hazard of T2D.

In Model 2, environmental attributes were jointly modeled as predictors of T2D, such that hazard ratios are independent. Higher residential greenness and green land cover remained protective factors for T2D, with hazard ratios attenuated by 11% and 53%, respectively, compared to Model 1 (e.g., for residential greenness, the HR of 0.936 was reduced to 0.943 in Model 2, where these refer to a 6.4 and 5.7% reduced rate of developing T2D per 0.1 NDVI, respectively). PM_10_ concentration was an independent risk factor for T2D, with the HR attenuated by 30% relative to Model 1. Both population density and intersection density were no longer significant predictors of T2D hazard, and HRs decreased by 100% and 27%, respectively. In contrast, the active commuting rate remained associated with T2D, and the HR increased by 22% relative to Model 1. Neither food environment indicator was associated with T2D hazard.

Schoenfeld residuals for environmental attributes from Model 2 indicated potential violations of the proportional hazards assumption for PM_10_, intersection density, active commuting, and food environment indicators. Thus, we examined Harrell’s rho coefficients (all were rho < 0.02) and plotted Schoenfeld residuals against time for environmental attributes. The results indicated only minor violations of the proportional hazards assumption. Additionally, we tested the interaction between time and the attributes that showed potential violations, finding only intersection density significantly interacted with time. The HR at time 0 was 1.047 (95% CI: 1.02, 1.08; *p* = 0.001) for intersection density and the interaction term indicated a weakening association over time (HR = 0.998, 95% CI: 0.997, 0.999, *p* = 0.005), equivalent to HR of 1.026 and 1.006 at year 10 and year 20, respectively.

In Models 3 and 4, we added two sets of covariates—namely, geographic variables (Model 3) and familial history of T2D (Model 4). HRs were similar for residential greenness and green land cover when accounting for these confounding variables. The association between PM_10_ concentration and T2D hazard remained significant but was attenuated by around 32% and 39% in Models 3 and 4, respectively, relative to Model 2. Greater intersection density was associated with higher T2D hazard in Models 3 and 4, where the HR more than doubled when adjusting for geographic variables and was attenuated when accounting for familial T2D history. A higher active commuting rate remained associated with reduced T2D hazard across models, although the HR decreased by 37% and 48% in Models 3 and 4 relative to Model 2. Neither food environment indicator was significantly associated with T2D hazard.

**Fig. 2.**
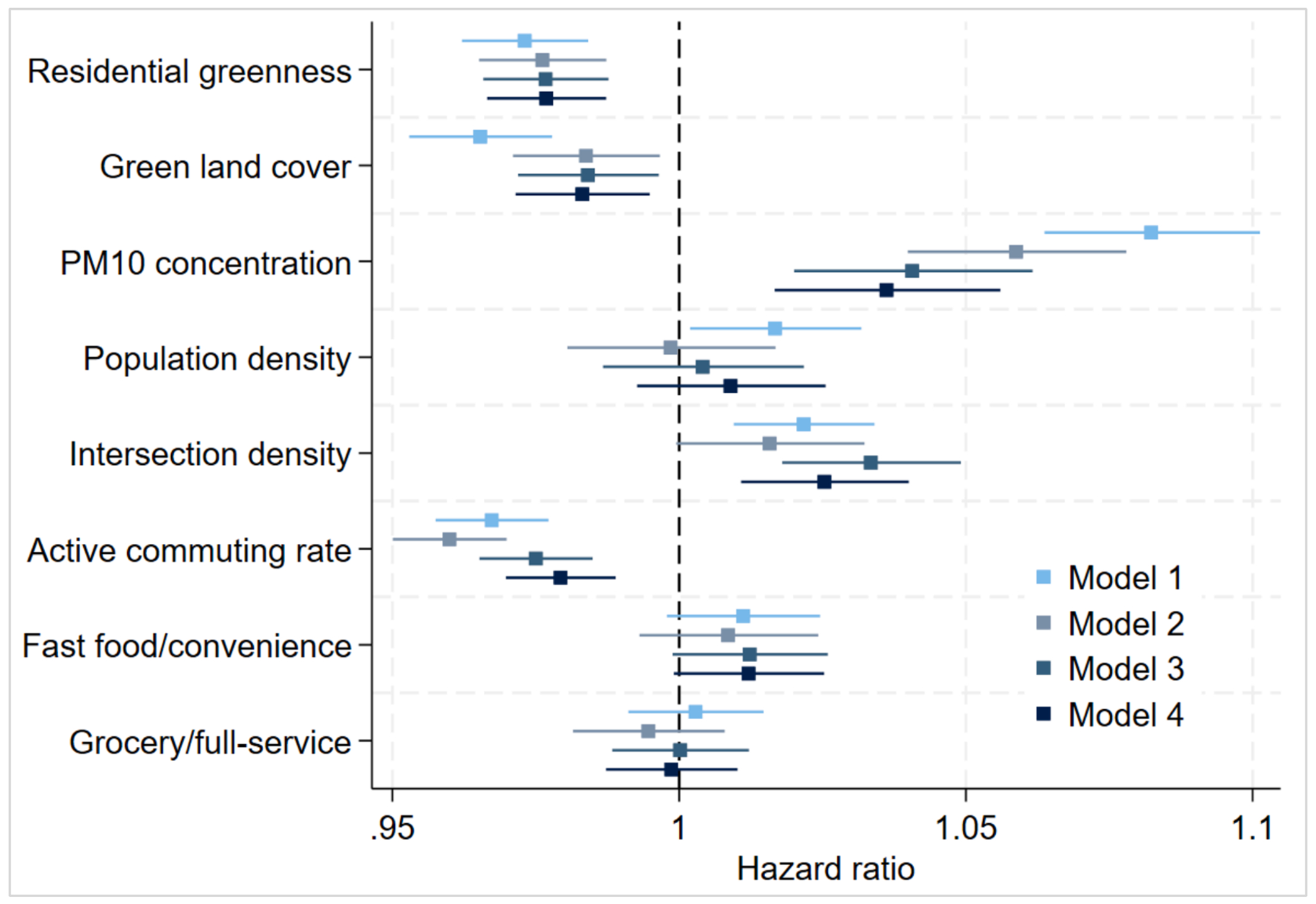
Coefficient plot of standardized associations between environmental attributes and hazard of type 2 diabetes mellitus under four different model specifications. *Note*. The hazard ratio and 95% confidence intervals are presented. Environmental attributes are standardized, such that hazard ratios are per standard deviation difference. All models are adjusted for individual and area covariates. Model 1 presents estimates for attributes when separately modeled, Model 2 presents the independent estimates when jointly modeling environmental attributes, Model 3 adds geographic covariates, and Model 4 adds familial history of T2D.

To facilitate comparison across attributes and provide effect sizes, we presented HRs for one standard deviation difference in environmental attributes (see Fig. 2). The figure indicates that residential greenness, PM_10_, and active commuting rate appeared to be the strongest and most consistent predictors of T2D hazard. However, we did not formally test differences due to the large number of potential comparisons.

Sensitivity tests generally confirmed the robustness of findings. First, when requiring three years of follow-up before observing T2D diagnosis, we found comparable HRs and model inferences (see Table S2 of Supplemental Material). Second, estimates were comparable when adjusting for period effects with identical model inferences (see Table S3 of Supplemental Material). Third, to examine if associations were sensitive to the scale of measurement, we modeled attributes at the level of census tracts instead of block groups (see Table S4 of Supplemental Material). Inferences for this model were similar, although associations for green land cover (OR = 0.989, 95% CI: 0.97, 1.00) and PM_10_ were not statistically significant (OR = 1.045, 95% CI: 1.00, 1.09). Finally, when fitting the model separately for the offspring and parent cohorts, residential greenness, intersection density, and the active commuting rate were associated with T2D hazard in a consistent direction (see Table S5 of Supplemental Material). However, more green land cover and fewer fast food/convenience stores were associated with lower T2D hazard only for the offspring cohort whereas PM_10_ related to higher T2D only for the parent cohort.

## 4. Discussion

The local environment for many US residents has been modified in potentially diabetogenic ways during the past half century due to automobile-oriented urban development patterns, declining residential greenness and urban tree cover, and the rise of fast food restaurants (Barrington-Leigh and Millard-Ball, 2015; Curtis et al., 2024; Karpyn et al., 2019; Nowak and Greenfield, 2018). In contrast, environmental regulations and technological progress have improved air quality with potential metabolic health benefits (Dominici et al., 2007; Yang et al., 2020). The present study shows that such local environmental changes may have influenced T2D risk for residents. In particular, block group-level residential greenness, green land cover, and active commuting rate were independently associated with reduced hazard of T2D among individuals. In contrast, higher PM_10_ concentration in block groups was independently associated with increased T2D hazard, while intersection density was a risk factor in some models. Overall, these findings support the need to incorporate T2D-related costs of environmental attributes in urban development planning and environmental regulations.

Findings for residential greenness and green land cover being protective factors are consistent with recent longitudinal studies (Doubleday et al., 2022; Tsai et al., 2021) and show that the associations are robust to extensive adjustment for co-exposures and covariates. Doubleday et al. (2022) used multiple waves of data from the Multi-Ethnic Study of Atherosclerosis to show that residential greenness was associated with reduced T2D incidence, but the study included only a narrow set of environmental covariates. Tsai et al. (2021) similarly reported an association between residential greenness and T2D incidence among more than 400,000 beneficiaries of the Taiwanese National Health Insurance program, yet the greenness exposure likely suffered from misclassification bias because it was defined in relation to the most-visited medical facility. Residential greenness and green land cover may influence diabetes risk through at least three general pathways—namely, reducing harm due to the ecosystem services of vegetation (e.g., lessening exposure to pollutants), restoring cognitive or psychological capacities (e.g., stress coping), and building capacities for healthful behaviors (e.g., physical activity, social cohesion) (Markevych et al., 2017). Our land cover measure includes green and other natural types, including water, barren, and wetland classifications, due to the semi-arid climate and potential health benefits of different types of natural landscapes (Li et al., 2023). Notably, in our study, estimates of the greenness-related benefits were attenuated when accounting for other environmental attributes, including air pollution. Prior research has shown that higher greenness may be an effect modifier or weaken the health effects of ambient air pollution, and that reduced air pollutants may mediate the benefits of greenness (as vegetation increases deposition and filtering of air pollutants) (Son et al., 2021). Overall, numerous mechanisms for the greenness-T2D link are plausible given the heterogenous uses of green amenities, with the possibility that such mechanisms also may vary based on other environmental attributes (Markevych et al., 2017).

Consistent with prior research (Liu et al., 2019; Yang et al., 2020), we found that exposure to higher ambient concentration of PM_10_ was associated with T2D incidence. In particular, 10 ug/m^3^ higher PM_10_ concentration was related to between 8.0 and 13.0% greater T2D hazard (from Models 2–4), similar to the meta-analyzed estimate of 11% (HR = 1.11) (Yang et al., 2020). Our study reduces the threat of omitted variable bias by adjustment for key covariates, including built environment attributes and both area and individual-level socioeconomic characteristics. Moreover, we report how the hazard ratio changes when adding geographic covariates. Yet, these estimates (i.e., Models 3 and 4) are likely conservative as distance to the interstate freeway, included as a covariate, proxies for a major source of air pollution. Mechanistic and genetic evidence for physiological pathways linking air pollution to T2D and glucose metabolism exists (Eze et al., 2016; Manisalidis et al., 2020).

Findings for block group walkability revealed a mixed picture, as greater intersection density was a risk factor for T2D incidence, after accounting for other geographic factors, whereas a higher active commuting rate consistently was a protective factor. Population density was not independently associated with T2D. Prior longitudinal studies have typically found that objectively measured neighborhood walkability is associated with reduced T2D risk (Chandrabose et al., 2019). However, walkability was generally measured as a composite in these studies, leading to a call for research on individual walkability dimensions as predictors of metabolic health to better inform urban design principles (Chandrabose et al., 2019). Our results indicate that greater intersection density, despite indicating a more connected street network, may increase the hazard of T2D for residents. This finding is consistent with another study in the region that found greater street connectivity was associated with higher obesity risk (Zick et al., 2009), an association that was stronger for non-low-income relative to low-income neighborhoods. In contrast, a study of Toronto residents reported that T2D prevalence rates decreased with greater street connectivity but this association was based on aggregate data and was only adjusted for age and sex (Glazier et al., 2014). Intersection density may have heterogenous impacts on T2D risk, with the benefits depending on factors such as the presence of walkable destinations (Glazier et al., 2014) and harms stemming from disamenities such as traffic-related air pollution or noise as well as crime perceptions (Dendup et al., 2019). For instance, in a national study of veterans, the association between walkability and diabetes risk was significant for suburban/small towns or rural areas, particularly with an above-median ratio of supermarkets to all food stores, but not urban areas (India-Aldana et al., 2022). Estimates for population density and intersection density also varied across models in the current study, indicating that point estimates and statistical inferences may be particularly susceptible to the correct model specification. Bivariate correlations indicated that higher population and intersection density were especially related to fewer green amenities, while also showing significant relationships with PM_10_ and food retailer availability. Further research is needed to closely identify the impact of specific walkability dimensions on health after adjustment for related co-exposures.

Similar to our findings on T2D, Zick et al. (2009) reported an association between a higher active commuting rate and reduced obesity risk. Although active commuting is infrequent in the study area, averaging around five percent of workers, we included this as a proxy measure for the use of active transportation (i.e., for work and non-work trips) likely relating to greater mixed-use development. Prior research has found that active commuting increases when employers are near housing and among individuals residing in the central city of a metropolitan area or in higher density housing (Cervero, 1996; Quinn et al., 2017). In other words, we expect that areas with higher rates of active commuting also are more likely to have diverse land use with residences, employers, and destinations intermingled, resulting in increased physical activity and spaces for social interactions. Research investigating the link between active commuting and T2D is warranted to identify the key contextual influences on active commuting and relevant T2D-related mechanisms. Notably, we found that estimates for intersection density and active commuting were attenuated when adjusting for familial history of T2D. Given the heritability of residential choice, particularly the likelihood to reside in urban settings (Duncan et al., 2012; Maxwell et al., 2021), our findings indicate that family health history may be an important control when estimating the association between neighborhood walkability and health.

Neither of our food environment measures was associated with T2D incidence, although the number of fast food/convenience stores was associated with greater T2D hazard among the offspring cohort. Prior longitudinal research similarly has found mixed evidence for density of fast food outlets predicting T2D risk (Gebreab et al., 2017; Mezuk et al., 2016). For instance, Gebreab et al (2017) reported that Black Americans experienced higher T2D incidence but not prevalence when residing in areas with more unfavorable food stores (i.e., convenience, fast food, dessert, and liquor stores). Our analysis categorized retailers into two broad types, which may mask heterogeneity across narrower food retailer types. A review by Black et al. (2014) found a higher share of studies on fast food restaurants reporting a significant association (i.e., nearly one-third) as compared to studies on convenience stores where nearly all reported a null association. This review also reported that around one-quarter of studies on grocery stores found an association with better dietary quality (Black et al., 2014), yet a meta-analysis reported a nonsignificant association between density of grocery stores and T2D (Dendup et al., 2018). Taken together, these findings suggest that although diet is a well-established determinant of T2D (Jannasch et al., 2017), food retailer availability alone may not be consistently related to T2D.

This study has notable strengths. These include an observation period of 24 years, a large sample size, time-varying measures for multiple environmental attributes, thorough covariate adjustment (i.e., co-exposures, individual and area-level socioeconomic characteristics, geographic correlates of attributes, and familial history of T2D), and use of multiple health data sources that likely captured the vast majority of T2D diagnoses for the cohorts. These strengths increase the credibility of the estimates for how environmental attributes relate to T2D incidence.

We also acknowledge several study limitations. First, due to data availability, we could not consider individual-level health behaviors (e. g., use of active transportation) that may mediate the relationship between environmental attributes and T2D. Second, we did not control for all possible environmental co-exposures and area covariates (e.g., crime perceptions, noise pollution), although correlates of such co-exposures were included (e.g., socioeconomic status, distance to freeway). Third, environmental measures were defined for block groups or tracts and did not include attributes of nearby areas or use neighborhoods that align with participant definitions of neighborhoods. Nonetheless, results were largely consistent across geographic scales, with the exception of green land cover. Fourth, the sample is derived from the urban core of Utah, limiting the generalizability to rural settings or other regions, although we expect the relationships to be similar in urban areas with similar levels of environmental exposures. Fifth, we could not directly account for neighborhood preferences that may relate to residential selection and bias associations between area attributes and T2D. However, prior research has shown that residential self-selection bias is small, that many individuals do not reside in areas congruent with their preferences, and that associations between neighborhood features and health generally persist after adjustment for urban amenity preferences (James et al., 2015; McCormack and Shiell, 2011). We also captured other determinants of residential selection (e.g., socioeconomic characteristics, geographic factors relating to the patterning of amenities), and included familial history of T2D to control for heritability of T2D and family influences on propensity to reside in an urban environment (Duncan et al., 2012; Maxwell et al., 2021; Willemsen et al., 2015). Finally, we modeled only the main effects of environmental attributes. Yet associations between local environmental attributes and T2D incidence are likely complex, and research is needed that examines interactions between environmental attributes and cross-level interactions involving area and individual characteristics (Eze et al., 2016). Future studies that integrate genetic predisposition or familial history of T2D with environmental exposures could identify subgroups most sensitive to the environment and inform targeted prevention efforts for individuals at elevated risk of T2D.

Extant literature, and this study, present consistent evidence for greenness, air pollution, and mixed land use being related to T2D (Bird et al., 2018). Improving the healthfulness of local environments therefore has potential for broad benefits to human health by shifting the curve or reducing risks at the population level (Rose, 1985), with the potential for major cost savings through T2D prevention. Conversely, although the emergence of effective drug therapies (i.e., GLP-1 agonists) for the treatment of T2D and obesity has been revolutionary and may eventually help reduce T2D rates in the population by effectively treating high-risk individuals (Kaplan et al., 2024; Rose, 1985), such individual-level interventions should not be at the cost of declining attention to environmental determinants.

## 5. Conclusions

This study provides longitudinal evidence in support of associations between multiple environmental attributes and T2D incidence. Among a large cohort in the urban core of Utah, our findings show that green amenities, air quality, and mixed land use development should be considered by local government and health organizations working to reduce rates of T2D. Such progress is likely to require multi-sector cooperation across healthcare, public health, and urban planning sectors, with community involvement, to create environments that encourage healthy behaviors while reducing environmental risks (Bird et al., 2018; Rydin et al., 2012). Recent neo-traditional and eco-urbanist development patterns emphasize walkability, brownfield redevelopment, and green infrastructure that may facilitate higher activity levels and social connections (Sharifi, 2016). This study shows that these forms of urban development may help reduce T2D risk among residents.

## Supplementary Material

1

## Figures and Tables

**Table 1:** Study variables and data sources.

Variable type	Variable definitions	Data sources; Years	Interval^a^
Outcome variable	• Type 2 diabetes (T2D) incidence	Healthcare Facility Database and Utah All Payers Claims Database, prepared by the Utah Department of Health and Human Services and obtained via the Utah Population Database; 1996–2019 and 2009–2019, respectively Multiple cause-of-death records from Utah vital statistics system; 1996–2019	Month/year
Environmental attributes	• Residential greenness (NDVI)	Landsat 5 and 8 from Google Earth Engine (Google, n.d.); 1995–1996, 2000–2001, 2005–2006, 2010–2011, 2015–2016	Five-year
	• Green land cover	Land Change Monitoring, Assessment, and Projection (United States Geological Survey, n.d.); 1992, 2001, 2011	Five-year
	• Particulate matter of 10 μm and smaller (PM_10_)	Center for Air, Climate and Energy Solutions (Kim et al., 2020); 1995–1996, 2000–2001, 2005–2006, 2010–2011, 2014–2015	Five-year
	• Population density	Decennial census estimates from the National Historical Geographic Information System (NHGIS) (Manson et al., 2022); 1990, 2000, 2010, 2020	Five-year
	• Intersection density	Topologically Integrated Geographic Encoding and Referencing data (US Census Bureau, 2021); 1992, 2000, 2010	Five-year
	• Active commuting rate (walk, bike, or public transit)	Decennial census and American Community Survey estimates from NHGIS (Manson et al., 2022); 1990, 2000, 2006–2010, 2013–2017	Five-year
	• Fast food or convenience stores • Grocery stores or full-service restaurants	D&B Million Dollar Directory (Dun and Bradstreet, 2010); 1990, 2000, and 2010	Five-year
Person-level covariates	• Age • Sex • Race and ethnicity • Latest educational attainment	Utah Population Database; 1990–2019 (Smith and Mineau, 2021)	Time-invariant
	• Marital status	Utah Population Database; 1990–2019 (Smith and Mineau, 2021)	Five-year
	• Charlson Comorbidity Index • Familial T2D history	Healthcare Facility Database and Utah All Payers Claims Database; 1996–2019 and 2009–2019, respectively Multiple cause-of-death records from Utah vital statistics system; 1990–2019 (only for familial T2D history)	• Five-year • Time-invariant
Area-level covariates	• Neighborhood socioeconomic status	Decennial census and American Community Survey estimates from NHGIS (Manson et al.,2022); 1990, 2000, 2006–2010, 2013–2017	Five-year
	• Residents identifying as non-Hispanic white, %	Decennial census estimates from NHGIS (Manson et al., 2022); 1990, 2000, 2010, 2020	Five-year
	• Longitude coordinates • Distance to city center • Distance to interstate freeway • County fixed effects	Prepared using geographic information system with point locations for city hall of the nearest metropolitan area primary city and the USA Major Highways layer from Esri	Time-invariant

a Interval at which variables are updated within analysis, with specific years for data sources shown in third column.

**Table 2:** Descriptive statistics for sample records by offspring and parent cohort.

Variables	Offspring cohort (*n* = 551,851)		Parent cohort (*n* = 357,878)	
	Mean ± SD	%	Mean ± SD	%
Age when becoming at risk	14.99 ± 5.72		41.18 ± 8.19	
Years at risk	20.35 ± 6.26		17.55 ± 6.97	
Female sex		49.05		53.49
Race and ethnicity				
Non-Hispanic (NH) White		87.37		88.77
Hispanic, any race		8.40		8.01
NH American Indian		0.18		0.13
NH Asian		0.39		0.67
NH Native Hawaiian or Other		0.20		0.17
Pacific Islander				
NH Black		0.23		0.30
NH Multiracial		2.44		1.79
Unknown		0.80		0.16
Latest educational attainment				
No high school diploma		5.10		9.29
High school diploma		19.56		33.80
Some college, no degree		25.58		29.40
Four-year college degree		15.42		15.25
Graduate/professional degree		6.29		10.29
Unknown		28.05		1.96
Marital status, time-varying				
Single		54.98		4.68
Married		40.94		81.04
Separated/divorced		4.04		13.85
Widowed		0.04		0.43
Charlson Comorbidity Index, time-varying	0.10 ± 0.42		0.21 ± 0.70	
Familial history of T2D	0.87 ± 1.04		0.77 ± 0.94	
T2D diagnosis during study		3.76		16.58

**Table 3:** Time-varying descriptive statistics for exposure to environmental attributes in study cohorts.

Block group attributes	1995	2000	2005	2010	2015	Maximum SMD
	M ± SD	M ± SD	M ± SD	M ± SD	M ± SD	
Socioeconomic status, SD	−0.23 ± 0.99	0.07 ± 0.98	0.08 ± 0.96	0.08 ± 1.00	−0.08 ± 1.02	0.31
Non-Hispanic White, %	89.66 ± 9.49	87.82 ± 11.48	85.52 ± 12.20	82.95 ± 13.64	78.93 ± 13.82	0.84
Residential greenness, range: 0–1	0.21 ± 0.04	0.20 ± 0.04	0.20 ± 0.04	0.20 ± 0.04	0.21 ± 0.04	0.33
Green land cover, %	6.93 ± 19.12	7.27 ± 19.99	8.28 ± 21.32	8.90 ± 22.20	9.91 ± 23.29	0.14
PM_10_, ug/m3	28.58 ± 3.22	28.08 ± 3.19	23.09 ± 3.07	21.05 ± 2.73	19.01 ± 2.56	2.00
Population density, 1000 s per sq km	1.70 ± 1.15	1.75 ± 1.24	1.70 ± 1.22	1.71 ± 1.21	1.71 ± 1.13	0.04
Intersection density, 10 s per sq km	1.53 ± 0.96	1.56 ± 0.96	1.56 ± 0.97	1.60 ± 0.98	1.53 ± 0.98	0.07
Active commuting rate, %	4.85 ± 4.86	4.54 ± 5.11	4.87 ± 5.35	5.16 ± 6.46	5.01 ± 5.92	0.11
Fast food/convenience stores, #	2.29 ± 2.64	2.80 ± 3.38	2.56 ± 2.77	2.40 ± 2.58	2.36 ± 2.61	0.18
Grocery/full-service restaurants, #	1.67 ± 1.97	2.11 ± 2.68	1.98 ± 2.50	1.90 ± 2.62	1.88 ± 2.65	0.18
Cohort members at each period:	*n* = 692,165	*n* = 685,905	*n* = 697,787	*n* = 667,912	*n* = 591,580	

*Note*. Food environment indicators are measured at the level of census tracts. Maximum standardized mean difference (SMD) between any two periods calculated using pooled standard deviation across periods.

**Table 4:** Cox proportional hazards model results for environmental attributes as predictors of type 2 diabetes incidence, *N* = 909,729 individuals with 15,240,973 person-years at risk.

	Model 1	Model 2	Model 3^a^	Model 4^a,b^

Environmental attributes	HR [95% CI]	HR [95% CI]	HR [95% CI]	HR [95% CI]
Residential greenness, 0.10	**0.936** [0.91, 0.96]	**0.943** [0.92, 0.97]	**0.945** [0.92, 0.97]	**0.945** [0.92, 0.97]
Green land cover, 20%	**0.967** [0.96, 0.98]	**0.985** [0.97, 1.00]	**0.985** [0.97, 1.00]	**0.984** [0.97, 1.00]
PM_10_, 10 ug/m3	**1.185** [1.14, 1.23]	**1.130** [1.09, 1.17]	**1.089** [1.04, 1.14]	**1.079** [1.04, 1.12]
Population density, 1000 s/sq km	**1.014** [1.00, 1.03]	0.999 [0.98, 1.01]	1.003 [0.99, 1.02]	1.008 [0.99, 1.02]
Intersection density, 10/sq km	**1.022** [1.01, 1.04]	1.016 [1.00, 1.03]	**1.034** [1.02, 1.05]	**1.026** [1.01, 1.04]
Active commuting rate, 10%	**0.943** [0.93, 0.96]	**0.931** [0.91, 0.95]	**0.957** [0.94, 0.97]	**0.964** [0.95, 0.98]
Fast food/convenience, 10 stores	1.041 [0.99, 1.09]	1.031 [0.98, 1.09]	1.045 [1.00, 1.10]	1.044 [1.00, 1.09]
Grocery/full-service, 10 stores	1.011 [0.97, 1.06]	0.979 [0.93, 1.03]	1.001 [0.95, 1.05]	0.995 [0.95, 1.04]

*Note*. Hazard ratios (HR) significant at *p* < 0.05 are shown in Bold font. Each attribute is separately modeled in Model 1. Attributes are jointly modeled in Models 2–4 and thus refer to independent associations. All models are adjusted for age, biological sex, race and ethnicity, marital status, maximum educational attainment, Charlson Comorbidity Index, block group socioeconomic status, block group non-Hispanic White percentage, and inclusion of records from All-Payers Claims database.

a Model adjusted for geographic variables: longitude coordinate, distance to city center, distance to interstate freeway, and county fixed effects.

b Model adjusted for T2D familial history.

## Data Availability

The data that has been used is confidential.

## Appendix A. Supplementary data

Supplementary data to this article can be found online at https://doi.org/10.1016/j.envint.2025.109870.
