# Supplementary material for: Local environmental attributes and type 2 diabetes: Green amenities, walkability indicators, and air pollution are associated with incidence
Source: Environ Int. Author manuscript; Available in PMC 2025 Dec 1. (PMC12667425; doi:10.1016/j.envint.2025.109870)
Supplement: 1 [file NIHMS2124780-supplement-1.docx]

Table S1. Bivariate correlations for time-varying block group environmental attributes across the five study periods (*n* = 6,228 block group-periods).

|  | 1. | 2. | 3. | 4. | 5. | 6. | 7. | 8. | 9. |
| --- | --- | --- | --- | --- | --- | --- | --- | --- | --- |
| 1. Socioeconomic status |  |  |  |  |  |  |  |  |  |
| 2. Non-Hispanic White | **0.69** |  |  |  |  |  |  |  |  |
| 3. Residential greenness | **0.46** | **0.39** |  |  |  |  |  |  |  |
| 4. Green land cover | **0.22** | **0.19** | **0.05** |  |  |  |  |  |  |
| 5. PM_10_ | **-0.23** | **0.01** | **-0.27** | **-0.27** |  |  |  |  |  |
| 6. Population density | **-0.37** | **-0.31** | **-0.20** | **-0.38** | **0.09** |  |  |  |  |
| 7. Intersection density | **-0.26** | **-0.27** | **-0.13** | **-0.49** | **0.09** | **0.73** |  |  |  |
| 8. Active commuting | **-0.36** | **-0.18** | **-0.25** | **-0.11** | 0.02 | **0.46** | **0.26** |  |  |
| 9. Fast food/convenience | **-0.22** | **-0.15** | **-0.28** | **-0.21** | **0.15** | 0.02 | **0.08** | **0.13** |  |
| 10. Grocery/full-service | **-0.25** | **-0.24** | **-0.28** | **-0.18** | **0.13** | **0.05** | **0.11** | **0.16** | **0.71** |

| *Note.* Bold font indicates statistical significance at *p* < .05. Food environment indicators are measured at the level of census tracts. |
| --- |

Table S2. Results for sensitivity analysis where individuals are excluded if T2D diagnosis is observed during the first three years of observation, *N* = 889,737 individuals with 13,146,017 person-years at risk.

|  | Model 4 |
| --- | --- |
| Environmental attributes | HR [95% CI] |
| Residential greenness, 0.10 | **0.946** |
|  | [0.92, 0.97] |
| Green land cover, 20% | **0.985** |
|  | [0.97, 1.00] |
| PM_10_, 10 ug/m3 | **1.091** |
|  | [1.05, 1.14] |
| Population density, 1000s/sq km | 1.008 |
|  | [0.99, 1.02] |
| Intersection density, 10/sq km | **1.027** |
|  | [1.01, 1.04] |
| Active commuting rate, 10% | **0.960** |
|  | [0.94, 0.98] |
| Fast food/convenience, 10 stores | 1.045 |
|  | [1.00, 1.10] |
| Grocery/full-service, 10 stores | 0.996 |
|  | [0.95, 1.04] |

*Note.* Hazard ratios (HR) significant at *p* < .05 are shown in Bold font. Model adjusted for age, biological sex, race and ethnicity, marital status, maximum educational attainment, Charlson Comorbidity Index, inclusion of records from All-Payers Claims database, block group socioeconomic status, block group non-Hispanic White percentage, tract geographic covariates (i.e., longitude coordinate, distance to city center, distance to interstate freeway), county fixed effects, and T2D familial history.

Table S3. Results for sensitivity analysis that includes adjustment for period fixed effects, *N* = 909,729 individuals with 15,240,973 person-years at risk.

|  | Model 4 |
| --- | --- |
| Environmental attributes | HR [95% CI] |
| Residential greenness, 0.10 | **0.948** |
|  | [0.92, 0.97] |
| Green land cover, 20% | **0.985** |
|  | [0.97, 1.00] |
| PM_10_, 10 ug/m3 | **1.084** |
|  | [1.04, 1.13] |
| Population density, 1000s/sq km | 1.007 |
|  | [0.99, 1.02] |
| Intersection density, 10/sq km | **1.026** |
|  | [1.01, 1.04] |
| Active commuting rate, 10% | **0.964** |
|  | [0.95, 0.98] |
| Fast food/convenience, 10 stores | 1.044 |
|  | [1.00, 1.09] |
| Grocery/full-service, 10 stores | 0.994 |
|  | [0.95, 1.04] |

*Note.* Hazard ratios (HR) significant at *p* < .05 are shown in Bold font. Model adjusted for age, biological sex, race and ethnicity, marital status, maximum educational attainment, Charlson Comorbidity Index, inclusion of records from All-Payers Claims database, block group socioeconomic status, block group non-Hispanic White percentage, tract geographic covariates (i.e., longitude coordinate, distance to city center, distance to interstate freeway), county fixed effects, T2D familial history, and period fixed effects.

Table S4. Results for sensitivity analysis where environmental attributes are at the level of census tracts instead of block groups, *N* = 909,729 individuals with 15,240,973 person-years at risk.

| Tract-level environmental attributes | Model 4 |
| --- | --- |
|  | HR [95% CI] |
| Residential greenness, 0.10 | **0.950** |
|  | [0.92, 0.98] |
| Green land cover, 20% | 0.989 |
|  | [0.97, 1.00] |
| PM_10_, 10 ug/m3 | 1.045 |
|  | [1.00, 1.09] |
| Population density, 1000s/sq km | 1.003 |
|  | [0.98, 1.03] |
| Intersection density, 10/sq km | **1.034** |
|  | [1.01, 1.06] |
| Active commuting rate, 10% | **0.919** |
|  | [0.90, 0.94] |
| Fast food/convenience, 10 stores | 1.030 |
|  | [0.98, 1.08] |
| Grocery/full-service, 10 stores | 0.999 |
|  | [0.95, 1.05] |

*Note.* Hazard ratios (HR) significant at *p* < .05 are shown in Bold font. Model adjusted for age, biological sex, race and ethnicity, marital status, maximum educational attainment, Charlson Comorbidity Index, inclusion of records from All-Payers Claims database, tract-level covariates (neighborhood socioeconomic status, non-Hispanic White percentage, longitude coordinate, distance to city center, and distance to interstate freeway), county fixed effects, and T2D familial history.

Table S5. Results for sensitivity analysis where models are stratified for the offspring (*n* = 551,851 individuals, 9,792,491 person-years) and parent cohorts (*n* = 357,878 individuals, 5,448,482 person-years).

|  | Offspring cohort | Parent cohort |
| --- | --- | --- |
| Environmental attributes | HR [95% CI] | HR [95% CI] |
| Residential greenness, 0.10 | **0.942** | **0.944** |
|  | [0.90, 0.98] | [0.91, 0.97] |
| Green land cover, 20% | **0.976** | 0.989 |
|  | [0.96, 1.00] | [0.98, 1.00] |
| PM_10_, 10 ug/m3 | 1.013 | **1.110** |
|  | [0.94, 1.09] | [1.06, 1.16] |
| Population density, 1000s/sq km | 0.999 | 1.013 |
|  | [0.98, 1.02] | [1.00, 1.03] |
| Intersection density, 10/sq km | **1.033** | **1.023** |
|  | [1.01, 1.06] | [1.00, 1.04] |
| Active commuting rate, 10% | **0.953** | **0.972** |
|  | [0.93, 0.98] | [0.95, 0.99] |
| Fast food/convenience, 10 stores | **1.145** | 1.010 |
|  | [1.05, 1.25] | [0.96, 1.06] |
| Grocery/full-service, 10 stores | 0.930 | 1.017 |
|  | [0.86, 1.00] | [0.97, 1.07] |

*Note.* Hazard ratios (HR) significant at *p* < .05 are shown in Bold font. Model adjusted for age, biological sex, race and ethnicity, marital status, maximum educational attainment, Charlson Comorbidity Index, inclusion of records from All-Payers Claims database, block group socioeconomic status, block group non-Hispanic White percentage, tract geographic covariates (i.e., longitude coordinate, distance to city center, distance to interstate freeway), county fixed effects, and T2D familial history.

STROBE Statement—Checklist of items that should be included in reports of ***cohort studies***

|  | Item No | Recommendation | | Section |
| --- | --- | --- | --- | --- |
| **Title and abstract** | 1 | (*a*) Indicate the study’s design with a commonly used term in the title or the abstract | | Abstract |
|  |  | (*b*) Provide in the abstract an informative and balanced summary of what was done and what was found | | Abstract |
| Introduction | | | | |
| Background/rationale | 2 | Explain the scientific background and rationale for the investigation being reported | | 1.1, 1.2 |
| Objectives | 3 | State specific objectives, including any prespecified hypotheses | | 1.2 |
| Methods | | | | |
| Study design | 4 | Present key elements of study design early in the paper | | 1.2 |
| Setting | 5 | Describe the setting, locations, and relevant dates, including periods of recruitment, exposure, follow-up, and data collection | | 2.2, 2.4 |
| Participants | 6 | (*a*) Give the eligibility criteria, and the sources and methods of selection of participants. Describe methods of follow-up | | 2.1 |
|  |  | (*b*) For matched studies, give matching criteria and number of exposed and unexposed | | NA |
| Variables | 7 | Clearly define all outcomes, exposures, predictors, potential confounders, and effect modifiers. Give diagnostic criteria, if applicable | | 2.3, Table 1 |
| Data sources/ measurement | 8* | For each variable of interest, give sources of data and details of methods of assessment (measurement). Describe comparability of assessment methods if there is more than one group | | 2.3, Table 1 |
| Bias | 9 | Describe any efforts to address potential sources of bias | | 2.3.6, 2.3.7, 2.4 |
| Study size | 10 | Explain how the study size was arrived at | | Figure 1 |
| Quantitative variables | 11 | Explain how quantitative variables were handled in the analyses. If applicable, describe which groupings were chosen and why | | 2.3 |
| Statistical methods | 12 | (*a*) Describe all statistical methods, including those used to control for confounding | | 2.4 |
|  |  | (*b*) Describe any methods used to examine subgroups and interactions | | 2.4 |
|  |  | (*c*) Explain how missing data were addressed | | 2.4 |
|  |  | (*d*) If applicable, explain how loss to follow-up was addressed | | 2.4 |
|  |  | (*e*) Describe any sensitivity analyses | | 2.4 |
| Results | | | |  |
| Participants | 13* | (a) Report numbers of individuals at each stage of study—eg numbers potentially eligible, examined for eligibility, confirmed eligible, included in the study, completing follow-up, and analysed | | Figure 1 |
|  |  | (b) Give reasons for non-participation at each stage | | 2, 2.4, Figure 1 |
|  |  | (c) Consider use of a flow diagram | | Figure 1 |
| Descriptive data | 14* | (a) Give characteristics of study participants (eg demographic, clinical, social) and information on exposures and potential confounders | | Tables 2 and 3 |
|  |  | (b) Indicate number of participants with missing data for each variable of interest | | Figure 1 |
|  |  | (c) Summarise follow-up time (eg, average and total amount) | | Table 2 |
| Outcome data | 15* | Report numbers of outcome events or summary measures over time | | Table 2 |
| Main results | 16 | | (*a*) Give unadjusted estimates and, if applicable, confounder-adjusted estimates and their precision (eg, 95% confidence interval). Make clear which confounders were adjusted for and why they were included | Table 4 |
|  |  |  | (*b*) Report category boundaries when continuous variables were categorized | NA |
|  |  |  | (*c*) If relevant, consider translating estimates of relative risk into absolute risk for a meaningful time period | Not reported |
| Other analyses | 17 | | Report other analyses done—eg analyses of subgroups and interactions, and sensitivity analyses | 3, Supplemental Material |
| Discussion | | | | |
| Key results | 18 | | Summarise key results with reference to study objectives | 4 |
| Limitations | 19 | | Discuss limitations of the study, taking into account sources of potential bias or imprecision. Discuss both direction and magnitude of any potential bias | 4 |
| Interpretation | 20 | | Give a cautious overall interpretation of results considering objectives, limitations, multiplicity of analyses, results from similar studies, and other relevant evidence | 4 |
| Generalisability | 21 | | Discuss the generalisability (external validity) of the study results | 4 |
| Other information | | | | |
| Funding | 22 | | Give the source of funding and the role of the funders for the present study and, if applicable, for the original study on which the present article is based | Acknowledgments |
